# Transcriptome comparison between pluripotent and non-pluripotent calli derived from mature rice seeds

**DOI:** 10.1038/s41598-020-78324-z

**Published:** 2020-12-04

**Authors:** Sangrea Shim, Hee Kyoung Kim, Soon Hyung Bae, Hoonyoung Lee, Hyo Ju Lee, Yu Jin Jung, Pil Joon Seo

**Affiliations:** 1 Department of Chemistry, Seoul National University, Seoul, 08826 Korea; 2 Plant Genomics and Breeding Institute, Seoul National University, Seoul, 08826 Korea; 3 Division of Horticultural Biotechnology, Hankyong National University, Anseong, 17579 Korea

**Keywords:** Plant sciences, Plant signalling

## Abstract

In vitro plant regeneration involves a two-step practice of callus formation and de novo organogenesis. During callus formation, cellular competence for tissue regeneration is acquired, but it is elusive what molecular processes and genetic factors are involved in establishing cellular pluripotency. To explore the mechanisms underlying pluripotency acquisition during callus formation in monocot plants, we performed a transcriptomic analysis on the pluripotent and non-pluripotent rice calli using RNA-seq. We obtained a dataset of differentially expressed genes (DEGs), which accounts for molecular processes underpinning pluripotency acquisition and maintenance. Core regulators establishing root stem cell niche were implicated in pluripotency acquisition in rice callus, as observed in *Arabidopsis*. In addition, KEGG analysis showed that photosynthetic process and sugar and amino acid metabolism were substantially suppressed in pluripotent calli, whereas lipid and antioxidant metabolism were overrepresented in up-regulated DEGs. We also constructed a putative coexpression network related to cellular pluripotency in rice and proposed potential candidates conferring pluripotency in rice callus. Overall, our transcriptome-based analysis can be a powerful resource for the elucidation of the molecular mechanisms establishing cellular pluripotency in rice callus.

## Introduction

Callus is a pluripotent cell mass, which can be produced from a single differentiated somatic cell^1,2^. Pluripotent callus can undergo de novo organ formation or embryogenesis, giving rise to a new organ or even an entire plant^2^. A balance of two phytohormones, auxin and cytokinin, underlies a two-step in vitro tissue culture^3^: incubation of tissue explants on auxin-rich callus-inducing medium (CIM) activates cell proliferation to facilitate callus formation^4^, whereas de novo shoot regeneration can be initiated by incubation on the cytokinin-rich shoot-inducing medium (SIM)^5,6^.

A particular emphasis has been placed on the callus formation process, because active cell proliferation drives pluripotency acquisition, which is a fundamental basis of plant regeneration^2^. Molecular mechanism underlying callus formation is starting to emerge. Accumulating evidence has shown that the CIM-derived callus resembles root primordium, regardless of origin of tissue explants^7–9^. In *Arabidopsis*, callus formation is initiated from the founder cell, such as pericycle (or pericycle-like) cells^8^. The founder cell undergoes asymmetric cell division and facilitates the acquisition of root primordium identity with the activation of genes including *WUSCHEL-RELATED HOMEOBOX 11* (*WOX11*) and *LATERAL ORGAN BOUNDARIES DOMAIN*s (*LBD*s)^9,10^. After acquiring root primordium characteristics, callus cells establish a regeneration competence via expression of root stem cell regulators, including *PLETHORA 1* (*PLT1*), *PLT2*, *SHORT-ROOT* (*SHR*), *SCARECROW* (*SCR*), *WOX5*, *WOX7*, and *WOX14*^7,8,11–13^. Consistently, *plt1plt2*, *scr*, and *wox5 wox7 wox14* mutants exhibit impaired de novo shoot regeneration possibly due to the failure of pluripotency acquisition^7,8,11,12^. It has been proposed that PLT and WOX-induced root stem cell identity is likely suspected as cellular nature of pluripotency^14–16^.

Several upstream regulators of the pluripotency factors have been identified. The *PLT3*, *PLT5*, and *PLT7* genes, which are induced upon incubation on CIM^11^, promote the acquisition of pluripotency through the induction of *PLT1* and *PLT2*^11^. Additionally, the root stem cell regulators are further coordinated by epigenetic modification. The HISTONE ACETYLTRANSFERASE OF THE GNAT/MYST SUPERFAMILY 1 (HAG1)/GENERAL CONTROL NONREPRESSED 5 (GCN5) protein binds to the promoters of the *WOX5*, *WOX14*, *SCR*, *PLT1*, and *PLT2* genes and deposits H3 acetylation (H3ac) to activate gene expression during callus formation^12,17^. The *hag1* mutant exhibits reduced plant regeneration with low expression of root stem cell genes^12^.

Although several molecular factors controlling cellular pluripotency during plant regeneration have been identified, the cellular feature of pluripotent callus cells has remained to be fully elucidated. Furthermore, a molecular signaling network involved in pluripotency acquisition has been investigated mostly in a model dicot plant *Arabidopsis*, and it is elusive whether the regulatory modules are conserved in monocot plants.

Cellular reprogramming is associated with changes in transcript accumulation^12,18–21^. Quantification of transcriptome can thus provide global insights into pluripotency acquisition during callus formation. In this investigation, we performed next-generation transcriptome sequencing to extend our understanding of the molecular mechanism controlling callus formation and pluripotency acquisition in rice. A comprehensive transcriptomic comparison between pluripotent and non-pluripotent calli, allowing to identify genes with potential roles in pluripotency establishment. In addition, based on the bioinformatics approach, we also proposed potentially relevant biological processes for pluripotency acquisition, including antioxidant metabolism. Overall, our findings suggest that cellular pluripotency is delicately regulated by readjusted hormonal and metabolic balance in plants.

## Results

### Transcriptome analysis identifies DEGs in pluripotent rice callus

**Figure 1:**
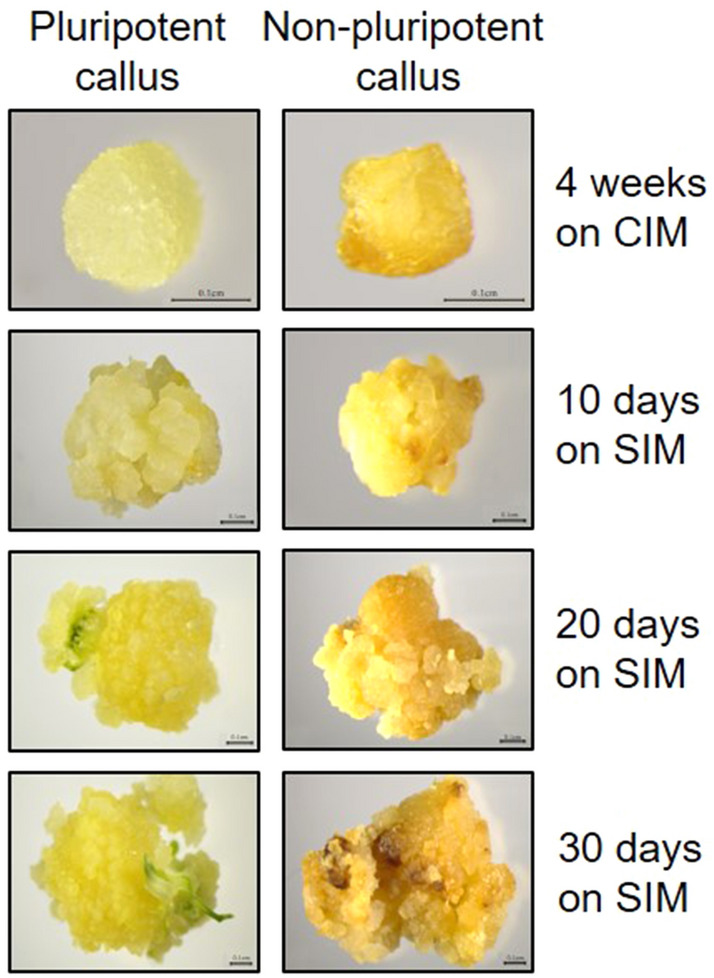
Morphological distinction between pluripotent and non-pluripotent rice calli. Left panels show pluripotent callus, and right panel shows non-pluripotent callus. Callus derived from mature rice seeds was incubated on auxin-rich callus-inducing medium (CIM) for 4 weeks. Then, calli were transferred to shoot-inducing medium (SIM).

While several molecular players involved in pluripotency acquisition have been identified in *Arabidopsis*, we wanted to know whether the gene networks are conserved in monocot rice plants. In addition, since we were able to distinguish pluripotent and non-pluripotent calli which were derived from mature rice seeds, we further tried to elucidate genetic candidates that regulate pluripotency acquisition in rice. Emerging calli could be sorted into pluripotent and non-pluripotent calli at 28 days after CIM incubation (DAC), based on their phenotypes (Fig. 1). Pluripotent calli with a globular-shaped compact cluster of yellowish cells had cellular competence for tissue regeneration, whereas non-pluripotent calli with semitransparent dark yellow color had a limited ability for de novo shoot regeneration (Fig. 1). The separation was reliable and reproducible, and the pluripotent and non-pluripotent calli divided at 28 DAC showed distinguishable regenerative potential during subsequent incubation on SIM (Supplementary Fig. S1 and Supplementary Table S1).

**Figure 2:**
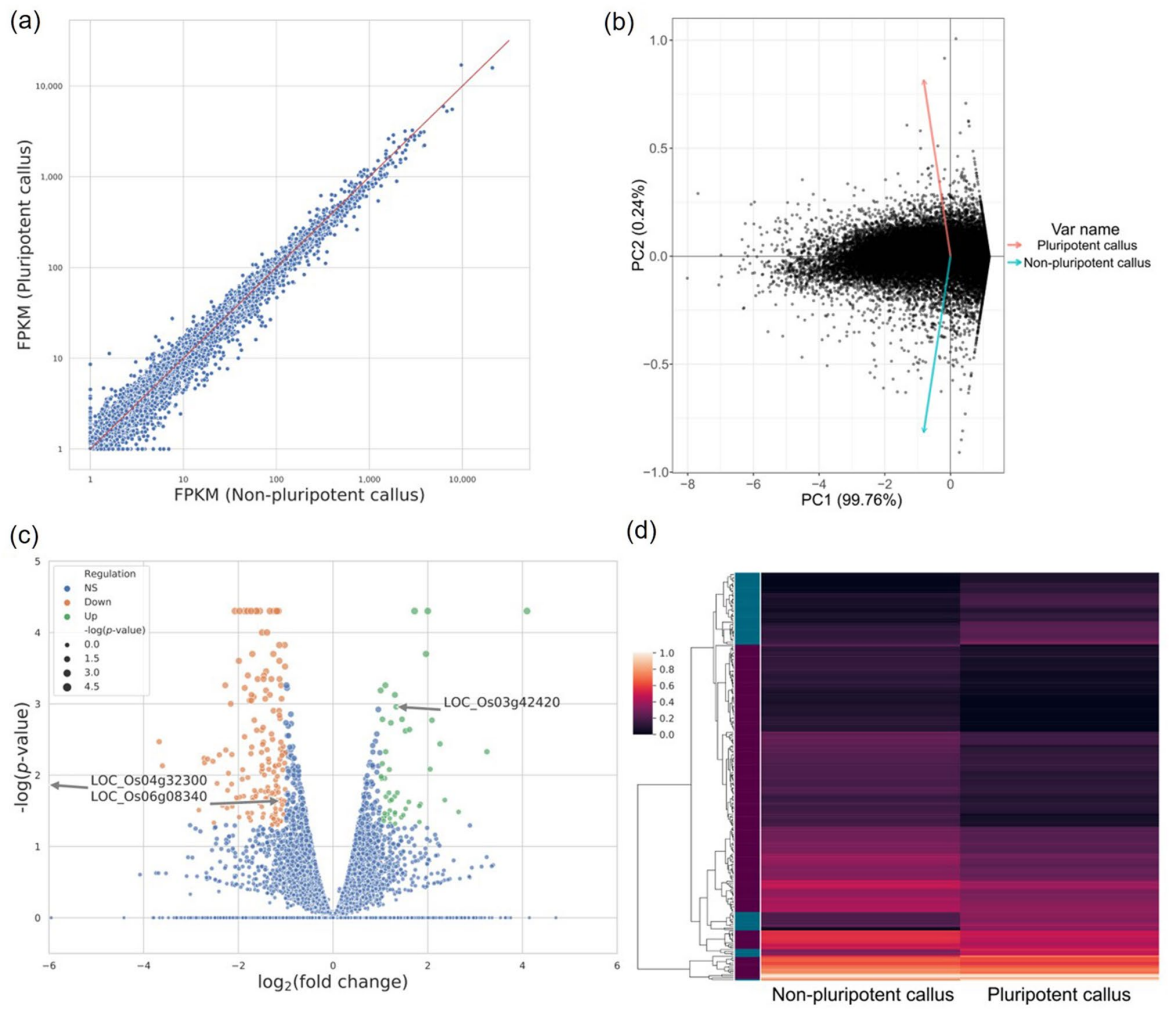
Comparison of global transcriptome between pluripotent and non-pluripotent calli. (**a**) Scatter plot comparing gene expression pattern in pluripotent and non-pluripotent calli. (**b**) PCA analysis of transcript abundance for two different types of callus mass. X- and y-axis indicate first and second principal components (PCs), respectively. Percent variance explained by each PC was presented along with axis labels. (**c**) Volcano plot showing significantly up-/down-regulated genes. X- and y-axis represent log_2_ transformed fold-change and log-transformed *p* value, respectively. (**d**) Heatmap showing expression profile of all DEGs. Hierarchical clustering was applied to cluster gene expression pattern in pluripotent and non-pluripotent calli. DEGs were distinguished by color map located in left side of dendrogram. Blue and purple bars represent up- and down-regulated DEGs, respectively.

We collected pluripotent and non-pluripotent calli at 28 DAC and extracted mRNAs to perform Illumina sequencing. Using the Illumina Hiseq-2000, we obtained 76,653,249 and 75,047,011 reads from pluripotent and non-pluripotent calli, respectively. Among these reads, 59,747,844 and 57,835,842 reads accounting for 77.9% and 77.1% of the total reads, respectively, were properly paired and mapped to the reference genome of japonica (MSU v.7.0; https://phytozome.jgi.doe.gov/pz/portal.html) (Table 1). Although transcriptome of the two different types of calli was considerably similar (Fig. 2a), principal component analysis (PCA) showed that the different callus types were also significantly separated along the first principal component, which explains 99.8% of the variability (Fig. 2b).

**Table 1:** Summary statistics of RNA-seq reads.

	Pluripotent callus Replicate 1	Pluripotent callus Replicate 2	Pluripotent callus Total	Non-pluripotent callus Replicate 1	Non-pluripotent callus Replicate 2	Non-pluripotent callus Total
No. of total reads	34,957,214	41,696,035	76,653,249	38,386,915	36,660,096	75,047,011
No. of mapped reads	34,957,214	41,696,035	76,653,249	38,386,915	36,660,096	75,047,011
No. of properly paired and mapped reads	27,753,148	31,994,696	59,747,844	29,488,630	28,347,212	57,835,842
Properly paired and mapped reads (%)	79.4%	76.7%	77.9%	76.8%	77.3%	77.1%

Based on the adopted cut-off (fold-change > 2 and *p* value < 0.05) (Fig. 2c), 60 up-regulated genes (Supplementary Table S2) and 184 down-regulated genes (Supplementary Table S3) in pluripotent callus relative to non-pluripotent callus were collected, which were validated by RT-qPCR analysis for randomly selected 23 DEGs (Supplementary Fig. S2 and Supplementary Table S4). Since a limited cell population may be related to pluripotency, observation of the moderate number of DEGs was reasonable. We performed a visual inspection of the hierarchical clustering results to identify major subgroups and clusters (Fig. 2d). Collectively, we successfully distinguished pluripotent and non-pluripotent calli to analyze transcriptome possibly related to cellular pluripotency.

### KEGG analysis reveals metabolic processes enriched in DEGs

**Figure 3:**
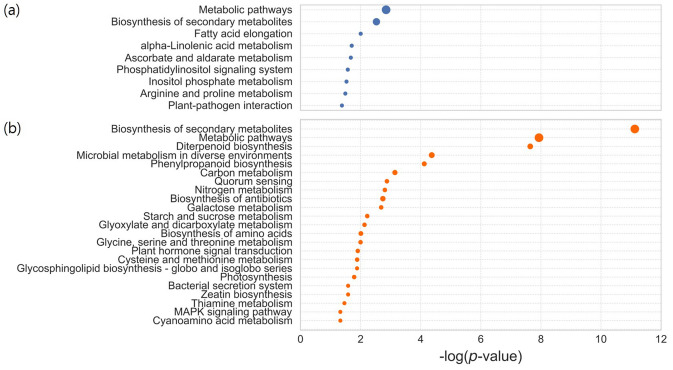
KEGG pathway enrichment analysis. Enrichment of KEGG terms of up-regulated genes (**a**) and down-regulated genes (**b**) in pluripotent callus relative to non-pluripotent callus was shown. X-axis indicates log-transformed *p* value. Size of circle represents difference between observed and expected ratio for a specific pathway. Statistical significance was tested by binomial test.

Changes in gene expression underlie differences in biological and metabolic functions between pluripotent and non-pluripotent calli. Gene ontology (GO) analysis of DEGs did not enrich a certain category. We thus alternatively explored the Kyoto Encyclopedia of Genes and Genomes (KEGG) terms that were enriched for up- and down-regulated genes in pluripotent calli. Within the group of up-regulated genes, we identified overrepresented KEGG categories for metabolic pathways (map01100), biosynthesis of secondary metabolites (map01110), fatty acid elongation (map00062), α-linolenic acid metabolism (map00592), and ascorbate metabolism (map00053) (Fig. 3a). This might reflect that pluripotent calli possibly promotes accumulation of secondary metabolites, such as ascorbic acid and antioxidants, as well as primary metabolites including fatty acids and lipids, compared with non-pluripotent calli (Fig. 3a), consistent with the fact that reactive oxygen species (ROS) and lipid molecules are closely associated with pluripotency acquisition in callus^22^.

In the case of the down-regulated genes, KEGG terms related to metabolic pathways (map01100), diterpenoid biosynthesis (map00904), phenylpropanoid biosynthesis (map00940), carbohydrate metabolic processes (map01200 and map00500), and hormone metabolism and signaling (map04075 and map04010) were enriched (Fig. 3b). The down regulation of these gene sets may be related to global alterations in plant metabolism and hormone signaling. Furthermore, consistent with the fact that callus tissues resemble to root primordium^7,8^, photosynthesis-related genes (map00195) were also included in this group.

### Primary and secondary metabolism and hormone signaling are related to pluripotency acquisition

**Figure 4:**
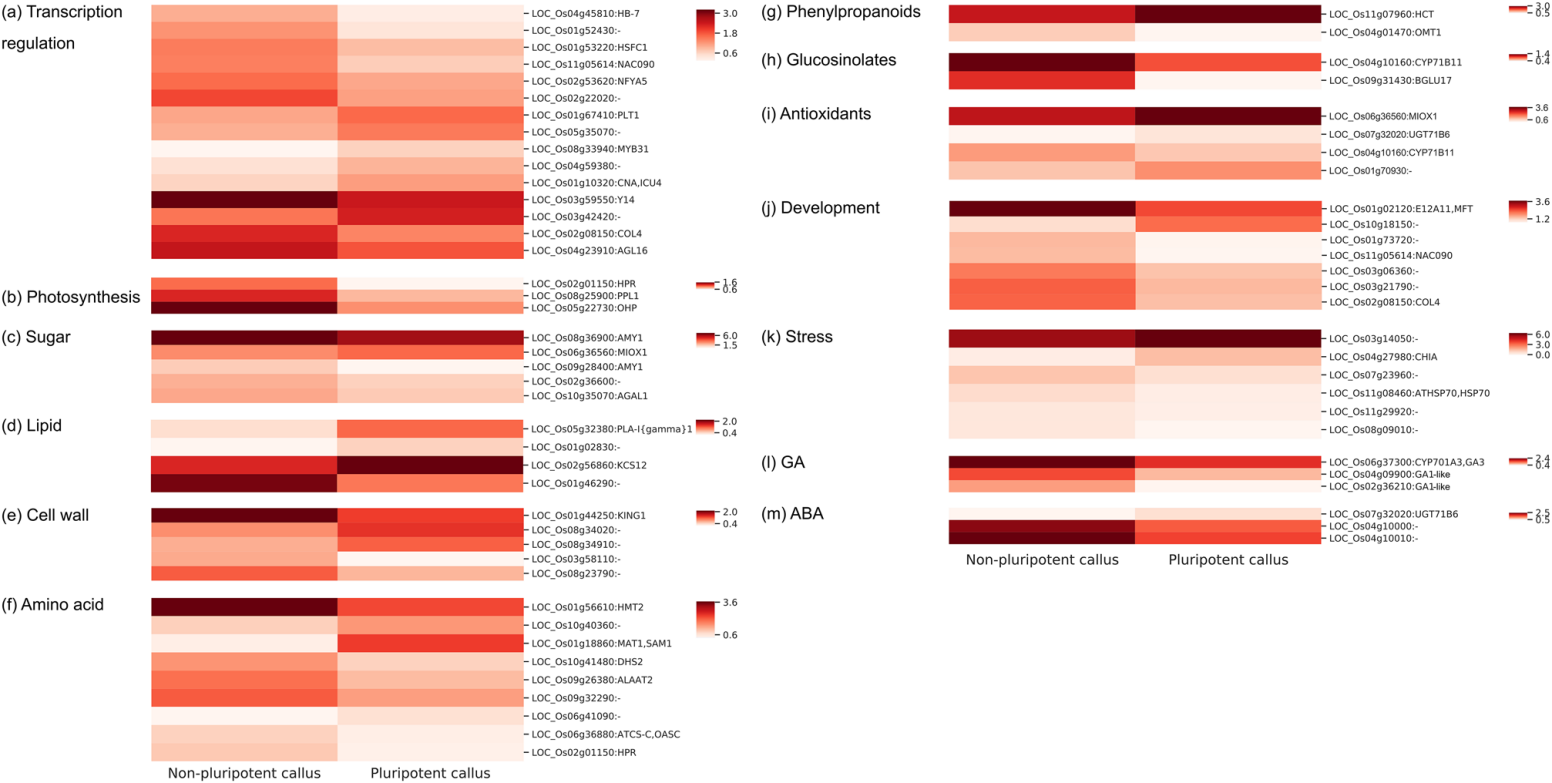
Expression patterns of selected DEGs. Transcript accumulation of DEGs related to transcriptional regulation (**a**), primary metabolism (**b–f**), secondary metabolism (**g**, **h**), antioxidant metabolism (**i**), developmental process (**j**), stress response (**k**), and hormone metabolism (**l**, **m**) is presented by heatmap. MSU identifiers and gene symbols are indicated.

We next wanted to know molecular factors underlying pluripotency establishment in rice callus. To this end, we first checked whether genes known to regulate cellular pluripotency were included in DEGs of pluripotent rice calli. As a result, the *PLT1* gene encoding an auxin-responsive transcription factor responsible for establishing root stem cell niche was up-regulated in pluripotent rice calli (Fig. 4a; Supplementary Table S2)^11,15^. Furthermore, a HSF transcription factor was also included (Fig. 4a), consistent with a possible role of HSFs in plant regeneration^23^. Other key transcription factors for various developmental processes were found in DEGs of pluripotent calli (Fig. 4a), implying that they might act as potential upstream regulators of pluripotency acquisition.

We expanded our analysis and examined more genes implicated in diverse aspects of primary and secondary metabolism in plants. Genes related to photosynthesis and primary metabolism for sugar, lipid, and amino acids were particularly enriched in our DEG lists (Fig. 4b–f), and they showed dynamic changes in pluripotent callus relative to non-pluripotent callus. Phenylpropanoid and glucosinolate metabolism genes were also differentially expressed (Fig. 4g,h), and most of which were down-regulated in pluripotent calli. Notably, a majority of antioxidant genes were up-regulated in pluripotent calli (Fig. 4i), as supported by KEGG analysis. In addition, several genes involved in developmental processes and stress responses were also changed in pluripotent calli (Fig. 4j,k), indicating that plant metabolic and developmental processes are massively reprogrammed during pluripotency establishment.

Callus formation and pluripotency acquisition are strongly associated with hormone metabolism and signaling^24^. To further inspect the relevance of hormone signaling in pluripotency acquisition in rice, a list of genes involved in hormone metabolism were investigated in DEGs. Although auxin and cytokinin signaling are known to play crucial functions in plant regeneration^3,21,24^, the genes related to auxin and cytokinin metabolism were negligibly influenced in pluripotent rice calli (see below). Instead, metabolic genes of gibberellic acid (GA) and abscisic acid (ABA) were particularly changed (Fig. 4l,m), suggesting that pluripotency acquisition in a limited cell population of callus might be conferred by intimate interactions between GA and ABA signaling.

Both pluripotent and non-pluripotent calli were generated by incubation on auxin-rich CIM, and they showed a comparable cell proliferation activity (Fig. 1). Since we compared both proliferating calli, genes involved in cell proliferation and cell cycle progression were negligibly influenced in pluripotent calli compared to non-pluripotent calli. It may also explain the observation that auxin and cytokinin signaling, which primarily affect cell division and proliferation, were not significantly changed. Since a limited cell population have distinguishable cellular features between pluripotent and non-pluripotent calli, the number of DEGs was small, and biological processes typically required for plant regeneration^25–28^ were limitedly enriched in the DEGs. Nonetheless, our analysis is still relevant in that the results enable to gain a new insight into the pluripotency acquisition in rice callus.

### Antioxidant activity is increased in pluripotent calli

Our RNA-seq analysis showed that many metabolic processes are regulated during callus formation, among which increased antioxidant activity in pluripotent calli was repeatedly suggested (Figs. 3a, 4i). In support, several previous studies have also shown that pluripotent stem cells contain higher levels of antioxidants in the maintenance of stemness^25,29–32^. We thus put our focus more on the relevance of antioxidant activity in pluripotent acquisition in rice callus.

We asked whether antioxidant levels are indeed increased in pluripotent calli. To this end, we measured levels of polyphenols, which includes several types of antioxidants such as phenolic acid, hydrolysable tannins and flavonoids with robust ROS-scavenging activity^33^, in pluripotent and non-pluripotent callus extracts. Polyphenol measurement revealed that total polyphenol levels of pluripotent calli were 1.2 times higher than those of non-pluripotent calli independently of callus incubation period (Table 2).

**Table 2:** Polyphenol content in pluripotent and non-pluripotent calli. Total content of polyphenolic compounds was determined according to Prussian Blue method (Hagerman and Butler^55^). Quercetin was used as a standard. Three biological replicates were averaged, and the data are represented as the mean ± standard deviation. DW, dry weight.

Callus incubation period on CIM (days)	Pluripotent callus (mg/g DW)	Non-pluripotent callus (mg/g DW)
28	48.3 ± 2.5	36.7 ± 1.6
56	62.8 ± 3.1	49.8 ± 2.6

The radical scavenging activity was further determined by the 2,2-diphenyl-1-picrylhydrazyl (DPPH) test. As a result, antioxidant activity was distinguishable between pluripotent and non-pluripotent calli. Pluripotent callus extract (EC_50_ = 1.1) had 12 times greater antioxidant activity compared to extracts from non-pluripotent (EC_50_ = 0.09) at 8 weeks after callus incubation (Table 3). These results indicate that enhanced antioxidant activity is critical for pluripotency maintenance in callus.

**Table 3:** Antioxidant activity in pluripotent and non-pluripotent calli extracts. Radical scavenging activity was determined using 2,2-diphenyl-1-picrylhydrazyl (DPPH). Butylated hydroxytoluene (BHT), 6-hydroxy-2,5,7,8-tetramethylchroman-2-carboxylic acid (Trolox), and ascorbic acid were used as positive controls. The EC_50_ value represents the concentration of antioxidant required to decrease the initial DPPH concentration by 50%. Three biological replicates were averaged, and the data are represented as the mean ± standard deviation.

Callus incubation period on CIM (days)	EC_50_ (mg/mL)	
	Pluripotent callus	Non-pluripotent callus
28	4.0 ± 0.8	3.4 ± 0.4
56	1.1 ± 0.07	0.09 ± 0.003
BHT	0.073 ± 0.012	
Trolox	0.006 ± 0.0009	
Ascorbic acid	0.123 ± 0.003	

### Network analysis identifies novel genes involved in pluripotency establishment in callus

Our dataset indicates that 244 genes were differentially expressed between pluripotent and non-pluripotent rice calli. The robustness of our transcriptome prompts us to query the expanded gene set for pluripotency acquisition. To this end, we decided to perform gene regulatory network (GRN) analysis to identify more candidate genes, whose expression is linked to core regulators of pluripotency acquisition. Based on a publicly-available GRN (RiceFREND; http:// https://ricefrend.dna.affrc.go.jp/), we first extracted the sub-network using *QUISCENT CENTER SPECIFIC HOMEOBOX* (*QHB*/*OsWOX5*) as the seed gene, which is known as a core regulator of pluripotency establishment^16,34,35^. In the subnetwork, *PLT1* and *PLT4*, the key players of plant regeneration, co-expressed with *QHB* (Supplementary Fig. S3). We also investigated the DEG genes in this sub-network containing *QHB* and *PLT*s and found that LOC_Os05g35070 was included as a potential regulator of pluripotent acquisition (Supplementary Fig. S3).

**Figure 5:**
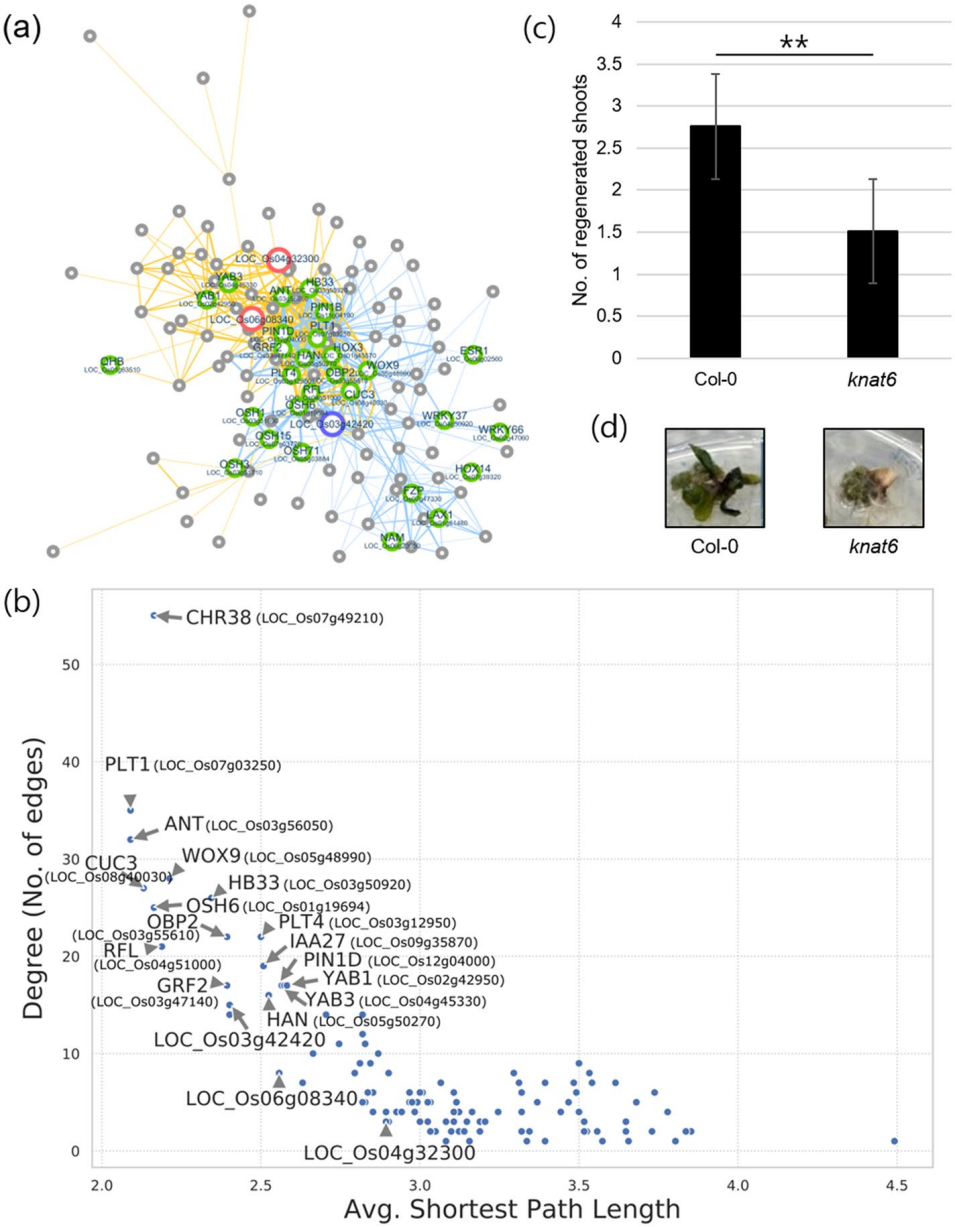
Putative pluripotency gene regulatory network (GRN) in rice. (**a**) Merged putative pluripotency GRN linking three DEGs and putative pluripotency regulators. Red and blue nodes indicate down- and up-regulated DEGs, respectively. Green nodes represent putative pluripotency regulators. Blue and orange edges indicate two different sub-networks identified by query DEGs, LOC_Os03g42420 and LOC_Os04g32300/LOC_Os06g08340, respectively. (**b**) Analysis of putative pluripotency GRN. X- and y-axis indicate average shortest path length and degree of each node (gene), respectively. (**c, d**) De novo shoot regeneration efficiency*.* The *Arabidopsis knat6* loss-of-function mutant (GK-478F03) was used. Leaf explant-derived calli incubated for 7 days on callus-inducing medium (CIM) were transferred to shoot-inducing medium (SIM). Photographs were taken at 21 DAS (days after incubation on SIM) (**c**). For quantification of shoot regeneration capacity, more than 45 calli at 21 DAS were used to count the number of regenerated shoots per callus. Three biological replicates were averaged (**d**). Bars indicate the standard error of the mean. Statistical significance is indicated by asterisk marks (ANOVA, **P < 0.01).

To obtain additional candidates regulating cellular pluripotency, we reciprocally extracted subnetworks using our DEGs. All DEGs in pluripotent callus were used as queries and a sub-network for each DEG was extracted. Among the input DEGs, only three genes, LOC_Os04g32300, LOC_Os06g08340, and LOC_Os03g42420, formed GRNs coexpressed with *QHB* and/or *PLT*s (Fig. 5a). The three subnetworks obtained could be merged (Fig. 5a; Supplementary Table S5), constructing a putative GRN related to pluripotency acquisition. Interestingly, the GRN basically contained core regulators of plant regeneration, such as *PLT1*, *PLT4*, *QHB*, *WOX9*, *ESR1*, and *CUC3*, suggesting that at least the three DEGs are potentially important for pluripotency acquisition in rice callus. From the putative pluripotency GRN, the top 10 hub genes were predicted based on the lowest average path length and higher connectivity (Fig. 5b): *CHR38*, *PLT1*, *ANT*, *CUC3*, *WOX9*, *HB33*, *OSH6*, *OBP2*, *PLT4*, and *RFL/LFY*. Among them, the potential role of *CHR38*, *ANT*, *HB33*, *OSH6*, and *RFL/LFY* have not yet been investigated for plant regeneration to date, and thus they can be considered as novel potential regulators of pluripotency acquisition.

To validate our prediction, we chose a candidate gene and analyzed the potential role in plant regeneration using *Arabidopsis* model system. The *OSH6* gene was of particular interest, because it is expressed at a confined pluripotent region of rice embryo, where shoot apical meristem (SAM) will be specified^36,37^. In addition, *OSH6* is also highly expressed in callus tissues (Supplementary Figs. S1 and S5). RT-qPCR analysis enabled to measure sensitive gene expression changes, compared with RNA-seq, and revealed that transcript accumulation of *OSH6* was significantly increased in pluripotent calli relative to non-pluripotent calli (Supplementary Fig. S4). Hence, we retrieved the *Arabidopsis* homolog of rice *OSH6* gene, *KNOTTED1-LIKE HOMEOBOX GENE 6* (*AtKNAT6*, At1g23380), based on phylogenetic analysis (Supplementary Fig. S6). To convince the *AtKNAT6* gene is the functional homolog of rice *OSH6* gene, we compared tissue-specific expression of Class I *KNOTTED1-LIKE HOMEOBOX* (*KNOX*) genes (*KNAT2* and *KNAT6*) closely related to the *OSH6* in the sub-clade (Supplementary Fig. S6) and confirmed that only *AtKNAT6*, but not *KNAT2*, is highly expressed in callus tissues similar to *OSH6* (Supplementary Fig. S5), suggesting that the *AtKNAT6* gene is a conserved homolog of rice *OSH6* gene possibly in the control of pluripotency acquisition during callus formation. We next obtained a *knat6* loss-of-function mutant (GK-478F03) and conducted in vitro tissue culture. The *knat6* mutant exhibited lower shoot regeneration efficiency with the reduced number of regenerated shoots (Fig. 5c and 5d), which was attributable to reduced pluripotency acquisition. Overall, we identified potential candidates implicated in pluripotency acquisition in rice callus, which are most likely associated with the stem cell specification and maintenance.

## Discussion

In vitro plant regeneration is driven by the exogenous application of phytohormones, auxin and cytokinin, and involves a two-step process: callus formation and de novo shoot regeneration^3^. In *Arabidopsis*, the hormone-induced callus is analogous to lateral root primordium^7–9^. The callus tissue further establishes root stem cell niche, through the expression of root stem cell regulators, including *PLT*s and *WOX*s^9,11,14–16,35^. Since PLT and WOX-induced root stem cell identity is a cellular nature of pluripotency^14–16^, they facilitate to form competence for tissue regeneration in callus. In support, only after expression of the pluripotency factors in callus, *PLT*s and *WOX*s, de novo shoot organogenesis can be initiated on SIM^11,12^. In this study, we compared the transcriptome of pluripotent and non-pluripotent calli to understand molecular networks involved in pluripotent acquisition in rice. Notably, molecular processes underlying pluripotent callus formation in rice are likely similar to those of dicot plants. *PLT*s were up-regulated in pluripotent callus relative to non-pluripotent callus in rice. Additionally, based on a putative GRN related to cellular pluripotency in rice (Fig. 5a), *WOX*s, *CUC*s and *ESR1*, which are known to be associated with cellular pluripotency in *Arabidopsis*^9,11,16,35,38–40^, were also involved in pluripotency acquisition in rice callus, indicating the similarity of hormone-induced callus of rice and *Arabidopsis*. Together, PLTs and WOXs are major determinants of cellular pluripotency across diverse plant species and can be used as key marker genes for pluripotency acquisition.

Several metabolic processes are associated with pluripotency establishment in rice callus. Photosynthetic processes significantly suppressed in pluripotent calli. Since callus resembles root primordium^7–9^, shoot developmental processes are essentially impaired in callus^20^. Consequently, primary metabolism was also globally influenced. In particular, sugar, amino acid, and nitrogen metabolism were suppressed during callus formation and pluripotency acquisition. We suspect that carbon and energy metabolism are substantially reprogrammed to optimize cellular pluripotency.

Notably, genes related to fatty acid metabolism and ROS homeostasis are enriched in upregulated DEGs of pluripotent calli. Several lipid species, such as prostaglandin E2, linoleic acid, and albumin‐associated lipids, are demonstrated as key metabolites for pluripotency establishment and maintenance in mammalian stem cells^41^. In addition, ROS acts either signaling molecules or detrimental agents inducing cell death^42,43^. ROS levels are delicately balanced in stem cell niche in plants, which is responsible for pluripotency acquisition^44,45^. Consistently, our study also showed that increased antioxidant activity in pluripotent calli is important for acquiring competence for de novo organogenesis. Overall, acquisition of cellular pluripotency is an active process that accompanies reprogramming of primary and secondary metabolism.

Since the pluripotent and non-pluripotent calli used in our study were incubated for same period of time on CIM, only a limited cell population distinguishes callus samples with different capacity of cellular pluripotency. Consistently, a small number of DEGs were obtained, and several key factors involved in auxin and cytokinin signaling and cell proliferation were unidentified from the DEGs. To understand biological relevance of 244 DEGs of pluripotent callus, coexpression network analysis was employed. Using a public database available for extracting coexpression network in rice, we could propose a putative GRN involved in cellular pluripotency (Fig. 5a). Several DEGs formed a GRN with core regulators of plant regeneration, such as *PLT*s, *WOX*s, and *CUC*s, and interestingly, they could be integrated into a larger coexpression network. Based on the results, several potential regulators can be suggested. The first group includes the DEGs that construct a gene networks with PLTs and WOXs: LOC_Os03g42420, LOC_Os04g32300, LOC_Os05g35070, and LOC_Os06g08340. The second group includes genes, which constitute a hub with high connectivity in the putative pluripotency GRN, but have not studied to date: *CHR38*, *ANT*, *HB33*, *OSH6*, and *RFL*. To validate our suggestion, we chose a candidate, *OSH6*, and confirmed its role in de novo shoot regeneration (Fig. 5c,d). The rice *OSH6* gene possibly regulates both cytokinin production and auxin responses^46–48^, linking cytokinin and auxin signaling in the control of pluripotency acquisition, although the detailed molecular mechanism should be elucidated in the future. Further, the remaining 8 potential candidates would also be associated with pluripotency acquisition and should be studied in the future to gain a comprehensive view of cellular pluripotency in rice.

Altogether, we propose a hypothesis how plant callus acquires pluripotency in rice. Based on the DEGs in pluripotent callus, we proposed molecular processes as well as putative GRNs involved in pluripotency acquisition. Our study provides candidates for evaluating the involvement of genes in pluripotency acquisition. It also provides a resource for comparative transcriptome analysis of plant regeneration in other species.

## Methods

### Callus induction, subculture, and regeneration

Mature seeds of Dongjin (*Oryza sativa* L., ssp. japonica) were soaked with 70% ethanol for 1 min, surface sterilized in 2.5% sodium hypochlorite supplemented with 2 drops of Tween20 for 20 min, and rinsed with sterile distilled water five times. Callus was induced on callus-inducing medium (CIM) supplemented with 2 mg/L 2,4-D, 0.3 mgL-1 casein, 0.5 mgL-1 L-proline, 0.5 mgL-1 L-Glutamine, 3% sucrose, and 0.3% gelrite and incubated for 4 weeks at 28 °C under continuous dark condition. Pluripotent and non-pluripotent calli were selected at 4 weeks after incubation on CIM and sub-cultured on shoot-inducing medium (SIM) containing 2 mgL-1 Kinetin, 0.2 mgL-1 NAA, 3% sucrose, and 0.8% agar for de novo shoot regeneration.

### RNA isolation and cDNA synthesis

Approximately 0.1 g (fresh weight) of pluripotent and non-pluripotent calli at 28 DAC were harvested respectively, and immediately frozen in liquid nitrogen for RNA isolation. Total RNAs were isolated using the RNeasy plant mini kit (Qiagen). Genomic DNA was eliminated by DNAse I (Invitrogen, Carlsbad, CA, USA) treatment, as recommended by the manufacturer. The purity of RNA samples was examined by use of the Agilent 2100 Bioanalyzer (Agilent Technologies). cDNA was synthesized from 300 ng RNA using the PrimeScript II First Strand cDNA Synthesis Kit MIX (Takara Bio, Japan) with oligo (dT) primers in a final volume of 20 μL according to the manufacturer’s instructions.

### Primer design and RT-qPCR conditions

Gene-specific primers for qRT-PCR analysis were designed using the primer 3.0 online tool (http://bioinfo.ut.ee/primer3/) according to the sequences of reference genes and a target gene. Primers were synthesized by the Bioneer Genomics Institute (Daejeon, Korea) with the following parameters: T_m_ values ranging from 50 to 65 °C, GC percent of 45–55%, primer lengths of 17–25 bp, and product lengths of 50–150 bp. RT-qPCR was carried out with the SYBR Green PCR Master Mix system (Bioneer, Daejeon, Korea) on an Applied Biosystems 7500/7500 Fast Real-time PCR System (ABI, CA, USA). The PCR amplification system and program were performed as described previously^49^. Three biological replicates were analyzed. The RefFinder online software was applied to further estimate the stability of reference genes. Relative gene expression levels were calculated using the 2^−ΔΔCt^ method^50^. All data were recorded as mean ± standard deviation and statistical significance was determined by SPSS (version 9.0 for Windows 98, SPSS Inc.). Primers used in RT-qPCR analysis were listed in Supplementary Table S6.

### Illumina sequencing and data analysis

For RNA-seq library construction, 1 μg of total RNA extracted from pluripotent calli and non-pluripotent calli incubated on CIM for 28 days. Since callus samples are heterogeneous and have significant variation in gene expression, we collected large amounts of samples (> 300 calli) for single biological replicate. Because of the sampling burden, we performed biological duplicates.

The total RNA was purified by DNase and mRNA purification kit. Then the purified mRNA samples fragmented in 94 °C for 8 min. Fragmented mRNAs were converted into cDNA using random primer. The cDNA samples were ligated by adapters using TruSeq RNA kit (Illumina, CA, USA) and amplified by PCR. Libraries were qualified using an Agilent 2100 Bioanalyzer (Agilent Technologies, CA, USA). To conduct paired end sequencing, libraries were subjected to Illumina HiSeq2000 platform (Illumina, CA, USA) for two biological replicates.

To identify high confidential DEGs, we mapped raw RNA-seq reads and quantified transcript abundance considering distribution of mate-pair inner distance. To this end, briefly, we preliminarily mapped the raw read sequences against primary reference transcriptome sequences of rice (MSU v.7.0; https://phytozome.jgi.doe.gov/pz/portal.html) using Bowtie2 aligner^51^. The mean and standard deviation of mate-pair inner distance were estimated for each sequencing library (Supplementary Table S7). Then, the raw reads were re-aligned against the rice reference genome (MSU v.7.0) considering mean and standard deviation of mate inner distance, and splice junction using Tophat2 and Bowtie2^51,52^. Transcriptome abundance was calculated using Cufflinks and Cuffquant along with application of –frag-bias-correct and –multi-read-correct options for precise quantification^53^. Statistical significance and fold change of each transcriptome was analyzed using Cuffdiff^53^. The differential expression analysis of two samples was performed using criteria, including the absolute value of log_2_ fold change ≥ 1 and *P* < 0.05, to ensure the significance of gene expression difference.

### KEGG pathway enrichment analysis

To conduct KEGG pathway enrichment analysis, the number of genes in a specific pathway was counted for gene group of interest and whole reference genome, respectively^54^. The statistical significance was calculated based on the binomial test comparing observed ratio of genes for specific pathway to expected ratio.

### Antioxidant activity analysis

All samples were homogenized with a mortar, dissolved in three volumes of methanol, and centrifuged at 12,000*g* for 10 min at room temperature. The supernatant was stored at − 80 °C until use for the analysis. The total content of polyphenolic compounds was measured according to the Prussian Blue method^55^ with several modifications. As a standard, Quercetin (Sigma Chemical Co., St. Louis, MO) was used.

Radical scavenging activity was measured according to a spectrophotometric method using an ethanol solution of DPPH^56^. As positive controls, Trolox (6-hydroxy-2,5,7,8-tetramethylchroman-2-carboxylic acid), BHT (butylated hydroxytoluene), and ascorbic acid were used. The EC_50_ values, defined as the amount of antioxidant necessary to decrease the initial DPPH concentration by 50%, were calculated. Three biological replicates were averaged, and statistical significance was determined by SPSS (version 9.0 for Windows 98, SPSS Inc.).

### Analysis of GRN for DEGs

To gain more insights into the acquisition of pluripotency from the DEGs, we searched GRNs carrying DEGs using RiceFREND database containing co-expression gene network which was constructed based on gene expression in various developmental stage and phytohormone treatments in rice^57^. All DEGs were queried in the database. The results were manually inspected whether the DEGs form a sub-network with key pluripotency regulators, such as *QHB*, *PLTs* and *CUC3*. The sub-networks link DEG with key molecular component of pluripotency were downloaded from the database and merged and visualized using Cytoscape^58^. Degree and average shortest path length of genes in merged network were analyzed using NetworkAnalyzer embedded in Cytoscape^59^.

### Phylogenetic and tissue-specific expression pattern analyses

To identify *Arabidopsis* orthologous gene of rice *OSH6* gene, we constructed phylogenetic tree using all *KNOTTED1-LIKE HOMEOBOX* (*KNOX*) genes in the *Arabidopsis* and rice genomes. The KNOX protein sequences from primary transcripts were retrieved and aligned using MUSCLE algorithm with default parameters^60^. An unrooted phylogenetic tree was constructed using MEGA X software based on the neighbor-joining algorithm with complete deletion for gaps and missing data treatment, the Jones-Taylor-Thornton (JTT) model for substitution model, and 1000 bootstrap replications for phylogeny test^61^.

In addition, to compare the tissue specific expression pattern of rice and *Arabidopsis* Class I *KNOX* genes, transcript accumulation values of the selected genes were downloaded from Rice Expression Database^62^ (http://expression.ic4r.org/index) and *Arabidopsis* eFP Browser^63^ (http://bar.utoronto.ca/efp/cgi-bin/efpWeb.cgi) to visualize the data.

### De novo shoot regeneration

Wild-type (Arabidopsis Col-0 ecotype) and *knat6* mutant (GK-478F03) seedlings were grown on Murashige and Skoog (MS) media at 22–23 °C under long-day condition (16-h light/8-h dark cycle) with fluorescent light (150 µmol photons/m^2^s). Leaf explants of third and fourth leaves from 2-week-old seedling were incubated on callus-inducing medium (CIM, B5 medium supplemented with 0.5 µg/ml 2,4-dichlorophenoxyacetic acid [2,4-D] and 0.05 µg/ml kinetin) for callus induction. The plates were incubated under 22 °C and continuous dark conditions for 2 weeks^64^. Calli incubated on CIM for 7 days were transferred to shoot-inducing medium (SIM, B5 medium supplemented with 0.9 µmol/l 3-indoleacetic acid, 2.5 µmol/l 2-isopentenyladenine). The plates were incubated at 25 °C and continuous light condition up to 3 weeks^65^.

## Supplementary information

Supplementary Information 1.

Supplementary Table S2.

Supplementary Table S3.

Supplementary Information 2.

## References

[1] Fehér A (2019). Callus, dedifferentiation, totipotency, somatic embryogenesis: What these terms mean in the era of molecular plant biology?. Front. Plant Sci..

[2] Ikeuchi M, Sugimoto K, Iwase A (2013). Plant callus: Mechanisms of induction and repression. Plant Cell.

[3] Skoog F, Miller CO (1957). Chemical regulation of growth and organ formation in plant tissues cultured in vitro. Symp. Soc. Exp. Biol..

[4] Shin J, Seo PJ (2018). Varying auxin levels induce distinct pluripotent states in callus cells. Front. Plant Sci..

[5] Cheng ZJ, Zhu SS, Gao XQ, Zhang XS (2010). Cytokinin and auxin regulates *WUS* induction and inflorescence regeneration in vitro in *Arabidopsis*. Plant Cell Rep..

[6] Che P, Gingerich DJ, Lall S, Howell SH (2002). Global and hormone-induced gene expression changes during shoot development in *Arabidopsis*. Plant Cell.

[7] Atta R (2009). Pluripotency of *Arabidopsis xylem* pericycle underlies shoot regeneration from root and hypocotyl explants grown *in vitro*. Plant J..

[8] Sugimoto K, Jiao Y, Meyerowitz EM (2010). *Arabidopsis* regeneration from multiple tissues occurs via a root development pathway. Dev. Cell.

[9] Liu J (2014). *WOX11* and *12* are involved in the first-step cell fate transition during de novo root organogenesis in *Arabidopsis*. Plant Cell.

[10] Feng Z, Zhu J, Du X, Cui X (2012). Effects of three auxin-inducible LBD members on lateral root formation in *Arabidopsis thaliana*. Planta.

[11] Kareem A (2015). *PLETHORA* genes control regeneration by a two-step mechanism. Curr. Biol..

[12] Kim J (2018). Epigenetic reprogramming by histone acetyltransferase HAG1/AtGCN5 is required for pluripotency acquisition in *Arabidopsis*. EMBO J..

[13] Sabatini S, Heidstra R, Wildwater M, Scheres B (2003). SCARECROW is involved in positioning the stem cell niche in the *Arabidopsis* root meristem. Genes Dev..

[14] Shimotohno A, Heidstra R, Blilou I, Scheres B (2018). Root stem cell niche organizer specification by molecular convergence of PLETHORA and SCARECROW transcription factor modules. Genes Dev..

[15] Aida M (2004). The *PLETHORA* genes mediate patterning of the *Arabidopsis* root stem cell niche. Cell.

[16] Forzani C (2014). WOX5 suppresses *CYCLIN D* activity to establish quiescence at the center of the root stem cell niche. Curr. Biol..

[17] Kornet N, Scheres B (2009). Members of the GCN5 histone acetyltransferase complex regulate PLETHORA-mediated root stem cell niche maintenance and transit amplifying cell proliferation in *Arabidopsis*. Plant Cell.

[18] Rymen B (2019). Histone acetylation orchestrates wound-induced transcriptional activation and cellular reprogramming in *Arabidopsis*. Commun. Biol..

[19] Ikeuchi M (2017). Wounding triggers callus formation via dynamic hormonal and transcriptional changes1[OPEN]. Plant Physiol..

[20] He C, Chen X, Huang H, Xu L (2012). Reprogramming of H3K27me3 is critical for acquisition of pluripotency from cultured *Arabidopsis* tissues. PLoS Genet..

[21] Gaillochet C (2017). Control of plant cell fate transitions by transcriptional and hormonal signals. eLife.

[22] Xu C, Cao H, Xu E, Zhang S, Hu Y (2018). Genome-wide identification of *Arabidopsis* LBD29 target genes reveals the molecular events behind auxin-induced cell reprogramming during callus formation. Plant Cell Physiol..

[23] Ikeuchi M (2018). A gene regulatory network for cellular reprogramming in plant regeneration. Plant Cell Physiol..

[24] Gaillochet C, Lohmann JU (2015). The never-ending story: From pluripotency to plant developmental plasticity. Dev. Camb. Engl..

[25] Lee K, Park O-S, Seo PJ (2016). RNA-Seq analysis of the *Arabidopsis* transcriptome in pluripotent calli. Mol. Cells.

[26] Lee K, Seo PJ (2018). Dynamic epigenetic changes during plant regeneration. Trends Plant Sci..

[27] Zhang K (2017). Differential deposition of H2A.Z in combination with histone modifications within related genes in *Oryza sativa* callus and seedling. Plant J. Cell Mol. Biol..

[28] Anzola JM (2010). Putative *Arabidopsis* transcriptional adaptor protein (PROPORZ1) is required to modulate histone acetylation in response to auxin. Proc. Natl. Acad. Sci. U. S. A..

[29] Ji J (2014). Antioxidant supplementation reduces genomic aberrations in human induced pluripotent stem cells. Stem Cell Rep..

[30] Rony IK (2015). Inducing pluripotency in vitro: Recent advances and highlights in induced pluripotent stem cells generation and pluripotency reprogramming. Cell Prolif..

[31] Suzuki YJ, Shults NV (2019). Antioxidant regulation of cell reprogramming. Antioxidants.

[32] Talkhabi M, Pahlavan S, Aghdami N, Baharvand H (2015). Ascorbic acid promotes the direct conversion of mouse fibroblasts into beating cardiomyocytes. Biochem. Biophys. Res. Commun..

[33] Urquiaga I, Leighton F (2000). Plant polyphenol antioxidants and oxidative stress. Biol. Res..

[34] Kamiya N, Nagasaki H, Morikami A, Sato Y, Matsuoka M (2003). Isolation and characterization of a rice *WUSCHEL*-type homeobox gene that is specifically expressed in the central cells of a quiescent center in the root apical meristem. Plant J..

[35] Haecker A (2004). Expression dynamics of *WOX* genes mark cell fate decisions during early embryonic patterning in *Arabidopsis thaliana*. Dev. Camb. Engl..

[36] Sentoku N (1999). Regional expression of the rice KN1-type homeobox gene family during embryo, shoot, and flower development. Plant Cell.

[37] Sentoku N, Sato Y, Matsuoka M (2000). Overexpression of rice OSH genes induces ectopic shoots on leaf sheaths of transgenic rice plants. Dev. Biol..

[38] Banno H, Ikeda Y, Niu Q-W, Chua N-H (2001). Overexpression of *Arabidopsis**ESR1* induces initiation of shoot regeneration. Plant Cell.

[39] Matsuo N, Makino M, Banno H (2011). *Arabidopsis**ENHANCER OF SHOOT REGENERATION* (*ESR*)*1* and *ESR2* regulate *in vitro* shoot regeneration and their expressions are differentially regulated. Plant Sci..

[40] Iwase A (2017). WIND1 promotes shoot regeneration through transcriptional activation of *ENHANCER OF SHOOT REGENERATION1* in *Arabidopsis*. Plant Cell.

[41] Wang L (2017). Fatty acid synthesis is critical for stem cell pluripotency via promoting mitochondrial fission. EMBO J..

[42] Tripathy BC, Oelmüller R (2012). Reactive oxygen species generation and signaling in plants. Plant Signal. Behav..

[43] Bhattacharjee S (2012). The language of reactive oxygen species signaling in plants. J. Bot..

[44] del Pozo JC (2016). Reactive oxygen species: From harmful molecules to fine-tuning regulators of stem cell niche maintenance. PLOS Genet..

[45] Zeng J, Dong Z, Wu H, Tian Z, Zhao Z (2017). Redox regulation of plant stem cell fate. EMBO J..

[46] Tsuda K, Ito Y, Sato Y, Kurata N (2011). Positive autoregulation of a KNOX gene is essential for shoot apical meristem maintenance in rice. Plant Cell.

[47] Sakamoto T (2006). Ectopic Expression of KNOTTED1-Like homeobox protein induces expression of cytokinin biosynthesis genes in rice. Plant Physiol..

[48] Hay A, Barkoulas M, Tsiantis M (2006). ASYMMETRIC LEAVES1 and auxin activities converge to repress BREVIPEDICELLUS expression and promote leaf development in *Arabidopsis*. Development.

[49] Huang X, Yang L, Jin Y, Lin J, Liu F (2017). Generation, annotation, and analysis of a large-scale expressed sequence tag library from *Arabidopsis pumila* to explore salt-responsive genes. Front. Plant Sci..

[50] Livak KJ, Schmittgen TD (2001). Analysis of relative gene expression data using real-time quantitative PCR and the 2(-Delta Delta C(T)) Method. Methods San Diego Calif.

[51] Langmead B, Salzberg SL (2012). Fast gapped-read alignment with Bowtie 2. Nat. Methods.

[52] Kim D (2013). TopHat2: Accurate alignment of transcriptomes in the presence of insertions, deletions and gene fusions. Genome Biol..

[53] Trapnell C (2012). Differential gene and transcript expression analysis of RNA-seq experiments with TopHat and Cufflinks. Nat. Protoc..

[54] Kanehisa M, Goto S (2000). KEGG: Kyoto encyclopedia of genes and genomes. Nucleic Acids Res..

[55] Hagerman AE, Butler LG (1994). Assay of condensed tannins or flavonoid oligomers and related flavonoids in plants. Methods Enzymol..

[56] Fraternale D, Giamperi L, Bucchini A, Ricci D (2009). Antioxidant activity of *Prunus spinosa* L. fruit juice. Ital. J. Food Sci..

[57] Sato Y (2013). RiceFREND: A platform for retrieving coexpressed gene networks in rice. Nucleic Acids Res..

[58] Shannon P (2003). Cytoscape: A software environment for integrated models of biomolecular interaction networks. Genome Res..

[59] Assenov Y, Ramírez F, Schelhorn S-E, Lengauer T, Albrecht M (2008). Computing topological parameters of biological networks. Bioinformatics.

[60] Edgar RC (2004). MUSCLE: A multiple sequence alignment method with reduced time and space complexity. BMC Bioinform..

[61] Kumar S, Stecher G, Li M, Knyaz C, Tamura K (2018). MEGA X: Molecular evolutionary genetics analysis across computing platforms. Mol. Biol. Evol..

[62] Xia L (2017). Rice Expression Database (RED): An integrated RNA-Seq-derived gene expression database for rice. J. Genet. Genom..

[63] Winter D (2007). An “electronic fluorescent pictograph” browser for exploring and analyzing large-scale biological data sets. PLoS ONE.

[64] Fan M, Xu C, Xu K, Hu Y (2012). LATERAL ORGAN BOUNDARIES DOMAIN transcription factors direct callus formation in *Arabidopsis* regeneration. Cell Res..

[65] Valvekens D, Montagu MV, Lijsebettens MV (1988). Agrobacterium tumefaciens-mediated transformation of *Arabidopsis thaliana* root explants by using kanamycin selection. Proc. Natl. Acad. Sci..

