# Supplementary material for: Transcriptome comparison between pluripotent and non-pluripotent calli derived from mature rice seeds
Source: Sci Rep. 2020 Dec 4;10:21257. doi: 10.1038/s41598-020-78324-z (PMC7719183; doi:10.1038/s41598-020-78324-z)
Supplement: Supplementary file 1 — Supplementary Information 1. [file 41598_2020_78324_MOESM1_ESM.docx]

**Supplementary Information**

**Transcriptome comparison between pluripotent and non-pluripotent calli derived from mature rice seeds**

**Sangrea Shim^1,2^, Hee Kyoung Kim^3^, Soon Hyung Bae^1^, Hoonyoung Lee^1^, Hyo Ju Lee^3^, Yu Jin Jung^3^, Pil Joon Seo^1,2^**

^1^Department of Chemistry, Seoul National University, Seoul 08826, Korea

^2^Plant Genomics and Breeding Institute, Seoul National University, Seoul 08826, Korea

^3^Division of Horticultural Biotechnology, Hankyong National University, Anseong 17579, Korea

**Address for correspondence**

Corresponding author: Pil Joon Seo, Ph.D.

Department of Chemistry, Seoul National University, Seoul 08826, Korea.

Phone: +82-2-880-7763

E-mail: pjseo1@snu.ac.kr

Corresponding author: Yu Jin Jung, Ph.D.

Division of Horticultural Biotechnology, Hankyong National University, Anseong 17579, Korea

Phone: +82-31-670-5101

E-mail: yuyu1216@hknu.ac.kr

**Authors’ email addresses**

Sangrea Shim, PhD sangreashim@gmail.com

Hee Kyoung Kim, Ms rudrud0329@naver.com

Soon- Hyung Bae, Mr tnsgud996@snu.ac.kr

Hoonyoung Lee, Ms gnsdud2@gmail.com

Hyo Ju Lee, Ms ju950114@naver.com

Yu Jin Jung, PhD yuyu1216@hknu.ac.kr

Pil Joon Seo, PhD pjseo1@snu.ac.kr

|  | Pluripotent callus | Non-pluripotent callus |
| --- | --- | --- |
| No. of subculture | 3 | 3 |
| No. of callus | 45 | 45 |
| No. of regenerated plants | 44 | 0 |

**Supplementary Table S1.** Regeneration potential of pluripotent and non-pluripotent calli derived from mature rice seeds.

**Supplementary Table S4.** Transcript accumulation of 23 randomly selected DEGs.

Transcript accumulation was analyzed by RT-qPCR analysis. Log_2_FC values for the selected genes in RNA‐seq and RT-qPCR were compared. FC, fold change.

| Gene ID | Annotation | Log_2_FC  (RNA-seq) | Relative transcript accumulation (2^-∆∆CT^) (RT-qPCR) | | | | | |
| --- | --- | --- | --- | --- | --- | --- | --- | --- |
|  |  |  | Replicate 1 | Replicate 2 | Replicate 3 | Average | Stdev | Log_2_ FC |
| LOC_Os01g18860 | S-adenosylmethionine synthetase 1 | **4.10** | 34.54 | 38.05 | 45.89 | 39.49 | 5.81 | **5.30** |
| LOC_Os04g27980 | chitinase A | **3.25** | 28.44 | 31.34 | 29.45 | 29.74 | 1.47 | **4.89** |
| LOC_Os08g33940 | myb domain protein 31 | **2.65** | 15.35 | 14.22 | 17.03 | 15.53 | 1.41 | **3.96** |
| LOC_Os01g16460 | Exostosin family protein | **2.36** | 2.69 | 2.57 | 2.58 | 2.62 | 0.07 | **1.39** |
| LOC_Os01g71860 | Glycosyl hydrolase superfamily protein | **2.26** | 2.85 | 2.95 | 3.41 | 3.07 | 0.30 | **1.62** |
| LOC_Os01g27230 | 12-oxophytodienoate reductase 2 | **2.09** | 2.60 | 2.55 | 2.77 | 2.64 | 0.12 | **1.40** |
| LOC_Os05g32380 | alpha/beta-Hydrolases superfamily protein | **2.05** | 5.13 | 5.21 | 4.82 | 5.05 | 0.20 | **2.34** |
| LOC_Os04g59260 | Peroxidase superfamily protein | **2.00** | 7.11 | 6.68 | 7.94 | 7.25 | 0.64 | **2.86** |
| LOC_Os10g18150 | crooked neck protein, putative / cell cycle protein, putative | **1.96** | 6.02 | 6.50 | 6.06 | 6.19 | 0.26 | **2.63** |
| LOC_Os01g02830 | Protein kinase superfamily protein | **1.85** | 2.23 | 2.53 | 2.64 | 2.47 | 0.21 | **1.30** |
| LOC_Os05g35070 | Protein of unknown function (DUF630 and DUF632) | **1.78** | 1.72 | 1.71 | 1.75 | 1.73 | 0.02 | **0.79** |
| LOC_Os03g42420 | AP2/B3-like transcriptional factor family protein | **1.34** | 1.61 | 1.57 | 1.38 | 1.52 | 0.13 | **0.60** |
| LOC_Os09g28400 | alpha-amylase-like | **-3.67** | 0.07 | 0.04 | 0.08 | 0.06 | 0.02 | **-4.00** |
| LOC_Os09g31430 | beta glucosidase 17 | **-2.65** | 0.07 | 0.07 | 0.07 | 0.07 | 0.00 | **-3.85** |
| LOC_Os02g09290 | cytochrome P450, family 71, subfamily B, polypeptide 24 | **-2.40** | 0.10 | 0.08 | 0.10 | 0.09 | 0.01 | **-3.46** |
| LOC_Os01g52430 | hAT dimerisation domain-containing protein | **-2.28** | 0.08 | 0.09 | 0.07 | 0.08 | 0.01 | **-3.71** |
| LOC_Os10g35040 | Leucine-rich repeat protein kinase family protein | **-2.26** | 0.47 | 0.37 | 0.45 | 0.43 | 0.05 | **-1.22** |
| LOC_Os02g36210 | Terpenoid cyclases/Protein prenyltransferases superfamily protein | **-2.16** | 0.31 | 0.25 | 0.30 | 0.29 | 0.03 | **-1.81** |
| LOC_Os05g47950 | UDP-Glycosyltransferase superfamily protein | **-2.14** | 0.35 | 0.32 | 0.44 | 0.37 | 0.06 | **-1.45** |
| LOC_Os01g56610 | homocysteine methyltransferase 2 | **-2.07** | 0.52 | 0.46 | 0.54 | 0.51 | 0.04 | **-0.97** |
| LOC_Os11g08460 | heat shock protein 70 | **-1.91** | 0.15 | 0.13 | 0.15 | 0.14 | 0.01 | **-2.80** |
| LOC_Os12g04424 | Calcium-dependent phosphotriesterase superfamily protein | **-1.87** | 0.62 | 0.46 | 0.76 | 0.62 | 0.15 | **-0.70** |
| LOC_Os06g08340 | Integrase-type DNA-binding superfamily protein | **-1.12** | 0.69 | 0.50 | 0.67 | 0.62 | 0.10 | **-0.69** |

**Supplementary Table S6.** Primers used in RT-qPCR analysis.

| Gene ID | Annotation | Product length  (start-end) | Forward primer | Reverse primer |
| --- | --- | --- | --- | --- |
| LOC_Os01g18860 | S-adenosylmethionine synthetase 1 | 121 (1052-1172) | TCATCATCAACCTCGACCTG | CACTTGAGGGGTTTCACCAC |
| LOC_Os04g27980 | chitinase A | 147 (757-903) | GCGAACAGCGGGTACGTC | CACGGCATGCTTTATCTTCT |
| LOC_Os08g33940 | myb domain protein 31 | 151 (778-928) | ACGAAGGACGAGGAGACC | AGAACTCACTCGGGTCCC |
| LOC_Os01g16460 | Exostosin family protein | 135 (246-380) | GAGGAGGTTGGCAAGCAG | GTCTGTTGACACCGCAAGTC |
| LOC_Os01g71860 | Glycosyl hydrolase superfamily protein | 112 (1770-1881) | CGGACAGATGGAGGCGTA | CTGATAGACCGGCGACTTGT |
| LOC_Os01g27230 | 12-oxophytodienoate reductase 2 | 126 (800-925) | TGTTCCTGGCCAACCCTGA | CATCCAGAAACGGGTAGTCC |
| LOC_Os05g32380 | alpha/beta-Hydrolases superfamily protein | 136 (1487-1622) | TGGTGAACAAGGCGTGCGA | TCGAGGTGCCAGCTGTGG |
| LOC_Os04g59260 | Peroxidase superfamily protein | 106 (874-979) | GCCAAGTCCATGGTCAAACT | CGAAGTCGACGAGGCTCT |
| LOC_Os10g18150 | crooked neck protein, putative / cell cycle protein, putative | 109 (2010-2118) | AGGAGGATGCGAGGAGTTCT | GCCACTGTTCTTCCATTCGT |
| LOC_Os01g02830 | Protein kinase superfamily protein | 136 (1631-1766) | GAGGAACCGTGGGCTACATA | GCATTTGGATCTGCATTCCT |
| LOC_Os05g35070 | Protein of unknown function (DUF630 and DUF632) | 145 (2468-2612) | ATGAGGAAGCCGTGAATCAA | CCACAACCATTGATTGGAA |
| LOC_Os03g42420 | AP2/B3-like transcriptional factor family protein | 150 (462-611) | TGACAAGAATGGCCATCACC | TCGTCCCCTTCCTTCTTCTC |
| LOC_Os09g28400 | alpha-amylase-like | 148 (1171-1318) | ATGGTCGACGAGAGGGTCAT | GCCGACCTTCTGGTTCTG |
| LOC_Os09g31430 | beta glucosidase 17 | 146 (1353-1498) | GGGCTACTTCACGTGGACAT | GCCTCTTGAGGAAGTCTTCGAT |
| LOC_Os02g09290 | cytochrome P450, family 71, subfamily B, polypeptide 24 | 140 (620-759) | AGCTCCTCCCGGAGATCG | GTCCACCAGCTCGTAGGATGT |
| LOC_Os01g52430 | hAT dimerisation domain-containing protein | 141 (48-188) | GACGATCGGGGAAGCGTA | GCGAGGTAGAGAGGGTCCTT |
| LOC_Os10g35040 | Leucine-rich repeat protein kinase family protein | 145 (1716-1860) | GGACATCCTCGACGTGGA | GATGTCGTCGATCATCCTGA |
| LOC_Os02g36210 | Terpenoid cyclases/Protein prenyltransferases superfamily protein | 140 (2255-2394) | GTGTCTACGGGAGCAGCAG | ATCCTCGAACAAAACCTTGG |
| LOC_Os05g47950 | UDP-Glycosyltransferase superfamily protein | 153 (1302-1454) | GATGCATTGGAAGCGAGAAG | ACCGGCCCATTCATAATCT |
| LOC_Os01g56610 | homocysteine methyltransferase 2 | 151 (824-974) | CGTGCGTCGGCAAGTGGA | ACTGCAGGGAAATCGTCGC |
| LOC_Os11g08460 | heat shock protein 70 | 140 (1537-1676) | AACGTTTCTGCCGAGCAC | TTTTGCTTGATCCGCTTCAT |
| LOC_Os12g04424 | Calcium-dependent phosphotriesterase superfamily protein | 150 (7-156) | TGGTGGAATATCATGTCAAGGA | TCCAGAACCTATGTGAATAACTGC |
| LOC_Os06g08340 | Integrase-type DNA-binding superfamily protein | 101 (330-430) | CAACAGCAACGACGACGACT | CATTGCCCAATGCGCCGG |

**Supplementary Table S7.** Summary statistics of mate inner distance for RNA-seq libraries used in this study.

|  | Pluripotent calli Replicate 1 | Pluripotent calli Replicate 2 | | Non-pluripotent calli  Replicate 1 | Non-pluripotent calli  Replicate 2 |
| --- | --- | --- | --- | --- | --- |
| Average (bp) | -22.36 | 0.00 | -1.70 | | -10.68 |
| Standard deviation (bp) | 60.63 | 68.69 | 70.42 | | 66.19 |


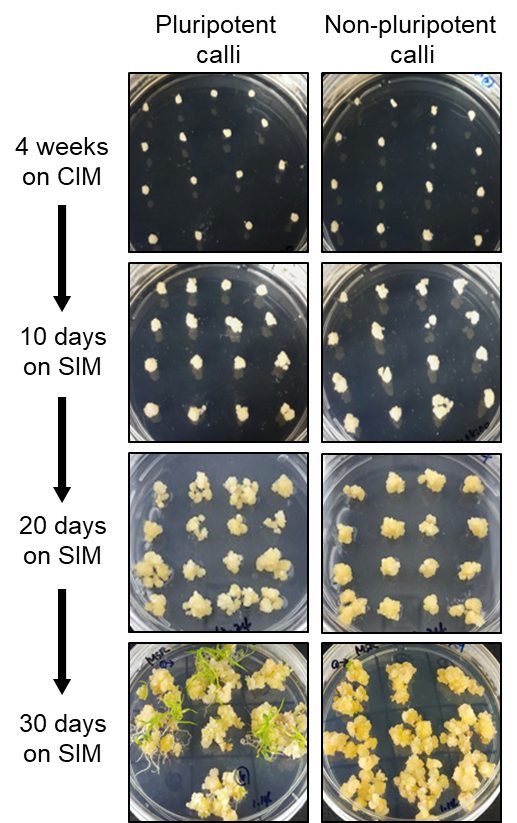


**Supplementary Figure S1.** Morphological characteristics of pluripotent and non-pluripotent calli derived from mature rice seeds. Callus derived from mature rice seeds was incubated on auxin-rich callus-inducing medium (CIM) for 4 weeks. Then, calli were transferred to shoot-inducing medium (SIM).

**
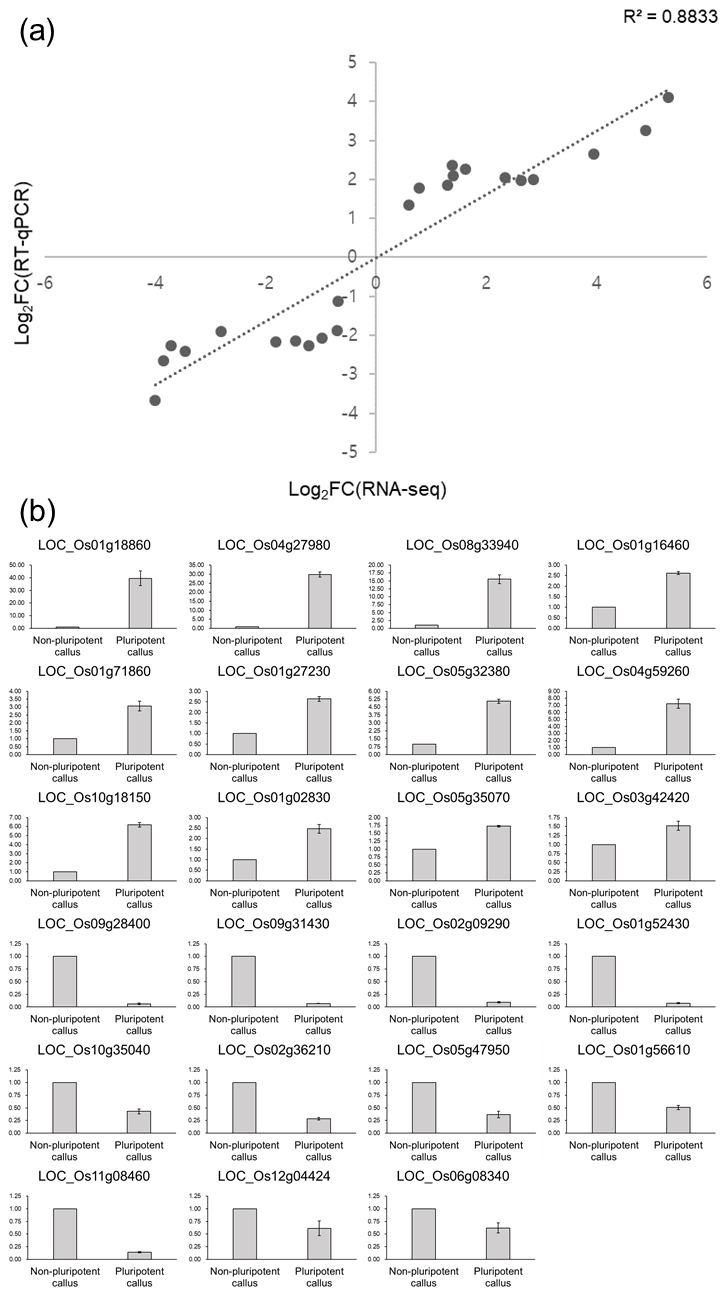
**

**Supplementary Figure S2.** Correlation between RNA-seq and RT-qPCR analyses.

(**a**) Correlation between RNA-seq and RT-qPCR data. Log_2_FC values for the selected 23 genes in RNA‐seq and RT-qPCR were compared (see Supplementary Table S4). FC, fold change. (**b**) RT-qPCR analysis. Transcript accumulation of selected 23 genes in pluripotent and non-pluripotent calli. Please see Supplementary Table S2 and S3.


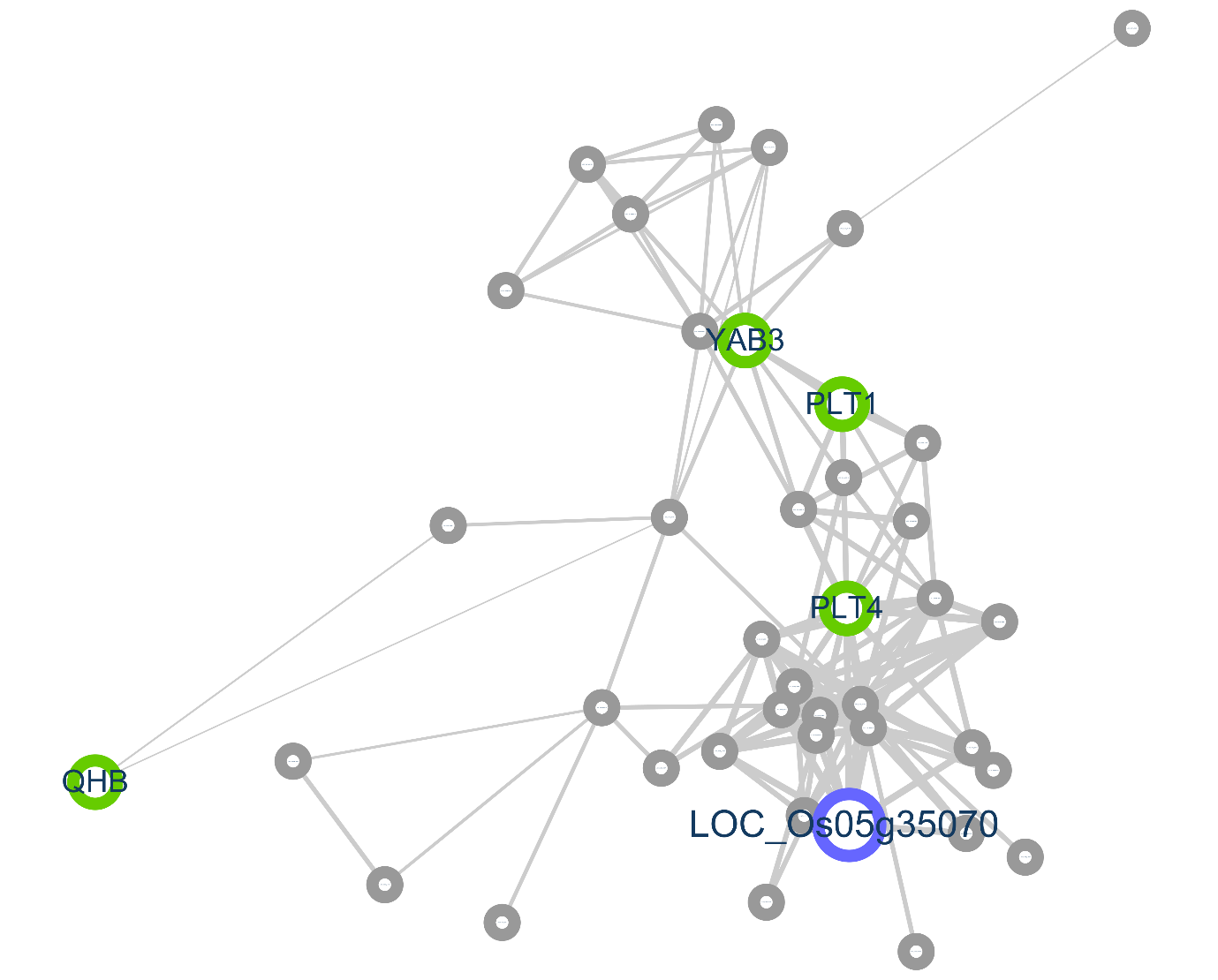


**Supplementary Figure S3.** Gene regulatory network extracted with *QHB*. Blue and green nodes represent up-regulated DEGs and core pluripotency regulators, respectively.

**
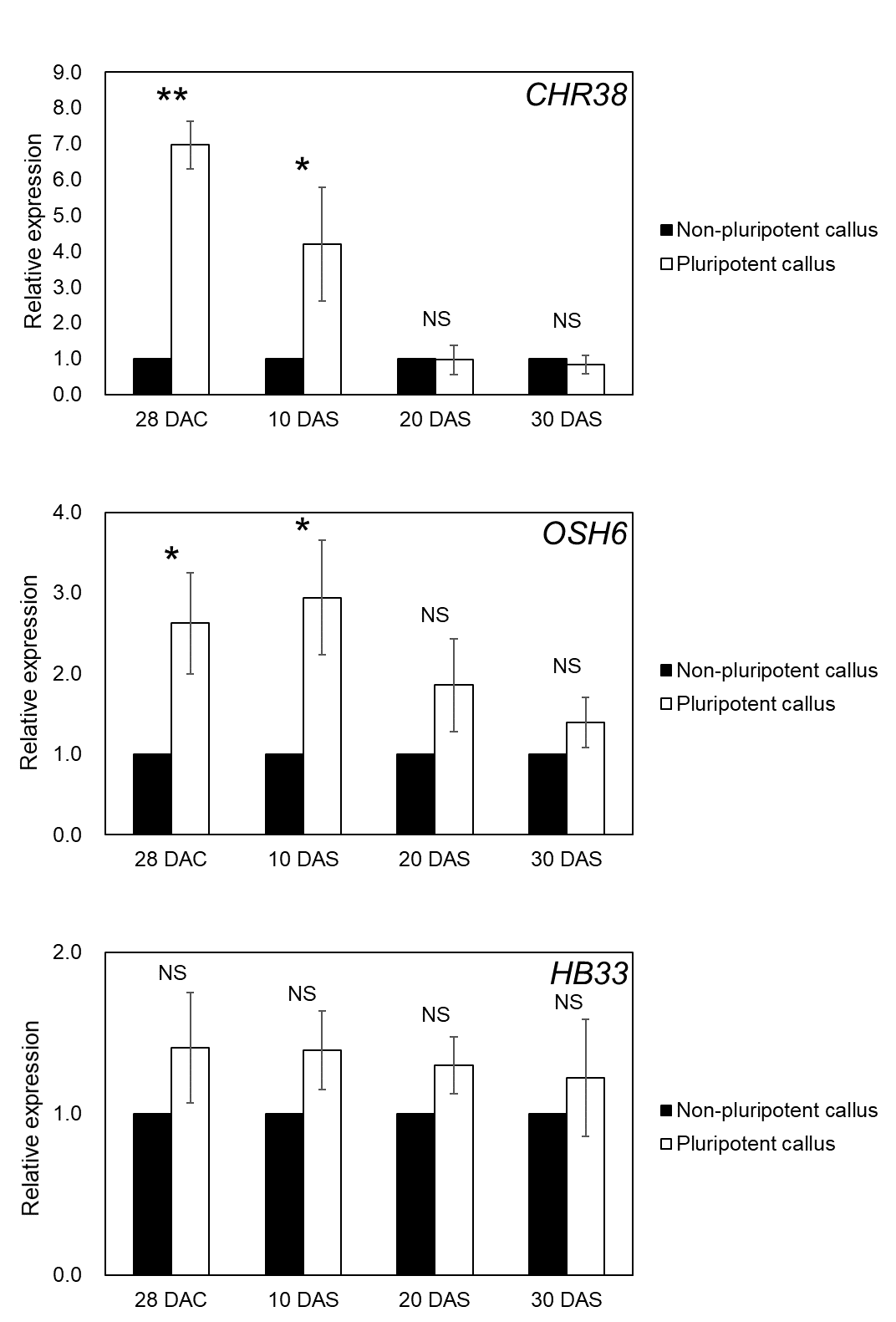
**

**Supplementary Figure S4.** Transcript levels of *CHR38*, *OSH6*, and *HB33* in pluripotent and non-pluripotent calli. RT-qPCR analysis was conducted to measure transcript accumulation. Statistical significance was determined by *t*-test (*, *P* < 0.05; **, *P* < 0.01; NS, not significant).


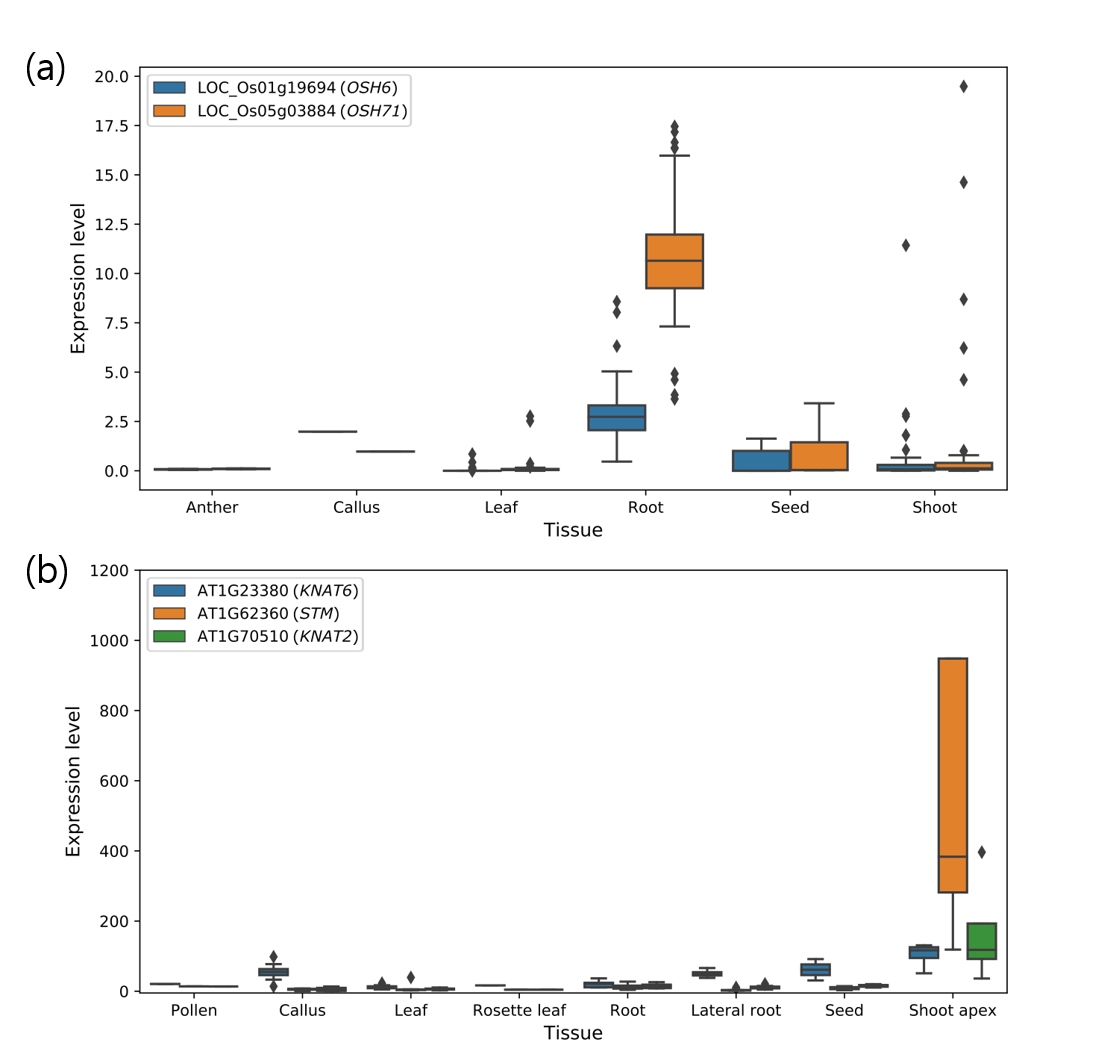


**Supplementary Figure S5.** Tissue-specific expression of selected Class I *KNOX* genes. Expression of selected *KNOX* genes from rice (**a**) and *Arabidopsis* (**b**) are shown. The data were retrieved from Rice Expression Database (http://expression.ic4r.org/index) for rice genes and eFP Browser (http://bar.utoronto.ca/efp/cgi-bin/efpWeb.cgi) for *Arabidopsis* genes.

**
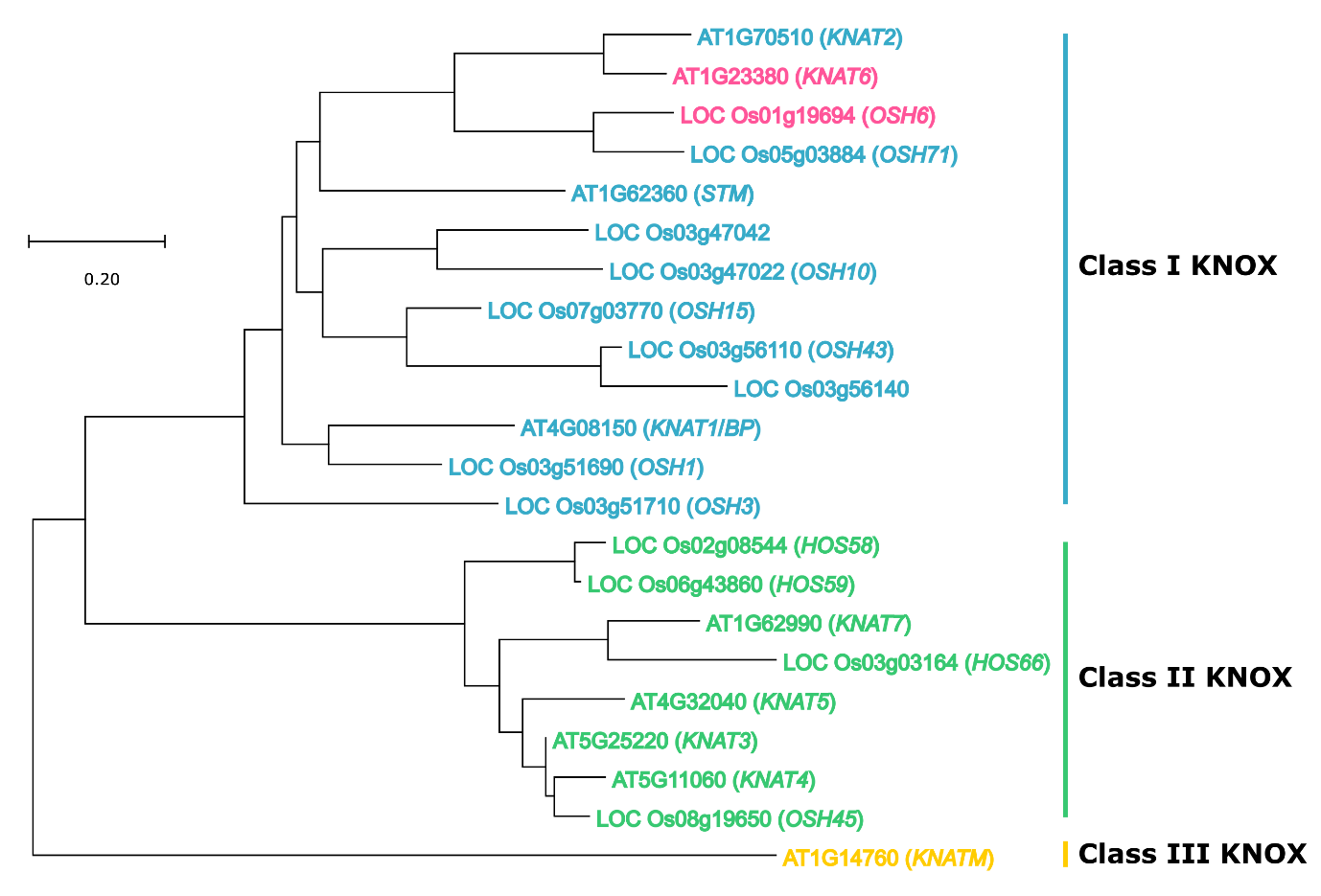
**

**Supplementary Figure S6.** An unrooted phylogenetic tree of KNOTTED-HOMEOBOX transcription factor genes in rice and *Arabidopsis* genomes. Phylogenetic tree was constructed based on the neighbour-joining (NJ) method in MEGA X. Members in the same class are shown in same color. The *OSH6* and *KNAT6* genes are shown in red.
